# Fingerprinting the Water and Vacancy Sites in Superhydrous Hematite: Neutron Scattering and First Principles Studies

**DOI:** 10.1063/4.0000944

**Published:** 2025-10-27

**Authors:** Si Athena Chen, Bryan C. Chakoumakos, James D. Kubicki, Matthias D. Frontzek, Luke L. Daemen, Yuanpeng Zhang, Jeffrey E Post, Peter J. Heaney

**Affiliations:** 1 Oak Ridge National Laboratory, Oak Ridge, TN, USA; 2 University of Texas at El Paso, El Paso, Texas, USA; 3 Smithsonian Institution, Washington, D.C., USA; 4 Penn State University, State College, PA, USA

## Abstract

Hematite (α-Fe_2_O_3_) is a low-cost, naturally abundant, and stable iron oxide with promising applications in energy applications such as photoelectrochemical water splitting, piezocatalysis, and microbial fuel cells. A superhydrous form, hydrohematite, incorporates significant structural water through cation vacancies, altering its physical and chemical properties. However, the positions and behavior of hydroxyl groups have not been fully characterized. In this study, we combined neutron powder diffraction, inelastic neutron scattering, pair distribution function analysis, and first-principles simulations to investigate the structure and dynamics of hydrogen in hydrohematite.

Rietveld refinement of neutron data revealed that hydroxyl incorporation and Fe vacancies expands the unit cell, particularly along the c-axis. The refined c/a ratio of hydrohematite was 2.737(2), slightly higher than the range of 2.731-2.734 in anhydrous hematite. Difference Fourier maps can account for half of the hydrogen positions, with the remainder too disordered to be apparent. Magnetic structure analysis revealed enhanced moments in hydrohematite, with a refined magnetic moment of 4.710(12) μB, compared to 3.968(163) μB in stoichiometric hematite. This slight increase is attributed to Fe deficiencies in the structure. Additionally, in-situ neutron diffraction indicated that the Morin transition, observed around 260K in the stoichiometric hematite, was suppressed in hydrohematite.

Combining inelastic neutron scattering and DFT calculations, we identified vibrational modes associated with hydroxyl motion at 800, 900, 1160, 1660, 2180, and 3450 cm^−¹^. Simulations indicated that the distinct 900 cm^−¹^ mode corresponds to out-of-plane OH deformation, while stretching modes between 2900–3500 cm^−¹^ confirm the presence of structural hydroxyls. DFT results also showed that hydrogen preferentially bonds with three of the six oxygens in an octahedral site, with calculated O-H bond lengths ranging from 0.97-1.02 Å and O–H⋅⋅⋅O hydrogen bond lengths between 1.63- 2.11 Å.

